# Core Decompression With Bone Marrow Aspirate Concentrate for Avascular Necrosis of the Femoral Head

**DOI:** 10.7759/cureus.78187

**Published:** 2025-01-29

**Authors:** Tiago Carvalho, Catarina Souto, Nuno Rodrigues, Roberto Couto, José Mesquita Montes

**Affiliations:** 1 Orthopaedics, Unidade Local de Saúde da Póvoa de Varzim/Vila do Conde, Póvoa de Varzim, PRT

**Keywords:** avascular necrosis of the femoral head, avascular osteonecrosis, bone marrow aspirate concentrate, core decompression, hip

## Abstract

Introduction: Avascular necrosis (AVN) of the femoral head is a complex and debilitating condition that predominantly affects a relatively young population. Core decompression of the femoral head is the primary surgical option for joint preservation, most commonly used in the early stages. However, clinical and radiological outcomes are variable in the literature. Biological therapies, such as bone marrow aspirate concentrate (BMAC), have also been used as adjuvants to complement this procedure. This study aims to evaluate the outcomes of this surgical intervention.

Methods: From 2012 to 2022, we conducted a retrospective study at a small Portuguese National Health Service hospital of all patients who underwent core decompression combined with the use of BMAC. Clinical data were reviewed, and telephone interviews were conducted, with functional scores completed and radiological criteria assessed. The primary outcome was preservation of the native hip at two years postoperatively.

Results: The series included 18 patients, totaling 24 affected hips. The average survival time after core decompression with BMAC was 14 months. The outcome was favorable in six (25%) hips, while 18 (75%) hips required a salvage arthroplasty. Patients with smaller preoperative lesions had more favorable outcomes than those with larger lesions.

Conclusion: The results of this study seem to confirm the importance of lesion size as a prognostic factor in the treatment of AVN lesions. Early stages alone do not guarantee surgical success.

## Introduction

Avascular necrosis (AVN) of the femoral head is a complex and debilitating condition, characterized by an interruption in intraosseous blood flow to this segment. This interruption leads to the formation of focal areas of ischemia, which evolve into necrosis [1]. The resulting increase in bone fragility causes bone deformity under repetitive loads, leading to progressive collapse and loss of the sphericity of the femoral head. These changes, if not identified and treated promptly, result in permanent joint destruction and degeneration [2]. AVN affects relatively young people, mostly men between 30 and 50 years old [3,4]. Although its etiopathogenesis has not been fully elucidated, it is associated with a variety of traumatic or atraumatic risk factors. These include femoral neck and head fractures, hip dislocation, smoking, alcoholism, corticosteroid use, diabetes, hemoglobinopathies, autoimmune diseases, vascular diseases, HIV infection, and exposure to radiotherapy or chemotherapy, among others [1,2].

Diagnosis is based on clinical assessment and imaging methods. Early stages may be asymptomatic, but the most frequent complaint is deep inguinal pain, in the anterior aspect of the thigh, with irradiation to the ipsilateral knee or, more rarely, to the gluteal region [1,2]. X-rays are typically taken in two views, using anteroposterior and frog-leg lateral projections. Magnetic resonance imaging (MRI) is the gold standard examination for diagnosing, classifying, and staging the disease. The most commonly used classifications are Ficat, Steinberg, and Association Research Circulation Osseous (ARCO) classifications [1-3].

Conservative treatment, consisting of rest, unloading or protected loading, and pharmacotherapy (with non-steroidal anti-inflammatory drugs and bisphosphonates), has a minimal role in AVN, applicable only in the earliest stages. Concerning surgical treatment, several options have been reported in the literature: core decompression, vascularized bone grafting, osteotomies of the proximal femur, or arthroplasty (resurfacing, partial, or total). Core decompression is most commonly used to treat early stages, and arthroplasty is the procedure of choice for later stages [1-5].

There is great interest in trying to preserve the femoral head whenever possible. To this end, the combination of core decompression and complementary biotechnology techniques has been advocated by some authors to enhance the stimulus for bone regeneration [5,6]. Some of these techniques include autologous bone marrow aspirate concentrate (BMAC), demineralized bone matrix, bone morphogenetic proteins (BMPs), or vascular endothelial growth factor (VEGF).

## Materials and methods

We conducted a retrospective study including patients who underwent surgical treatment for AVN, with core decompression in combination with the use of BMAC, in a single hospital of the Portuguese National Health Service, from 2012 to 2022. The inclusion criteria were patients aged between 18 and 60 with ARCO stages I, II, and III AVN. Exclusion criteria included patients with a previous hip procedure and patients who had not completed at least one year of follow-up. In total, 18 patients (24 hips) with an average follow-up of 25 months (12-41) were included. The clinical data from the electronic file were consulted, and telephone interviews were carried out to complete the information required for the study.

The percentage of preserved native hips (without the need for arthroplasty) at two years was defined as the primary outcome. Additional variables analyzed included demographic data, lesion laterality, associated risk factors, staging (ARCO classification), lesion extent (modified Kerboul angle and Kim and Koo index), native hip survival time after decompression, and clinical results in patients with a favorable primary outcome (using the Postel-Merle d'Aubigné functional score).

The radiological assessment of necrosis angles was initially carried out by a single observer, with subsequent analysis and validation by a second observer. The work was submitted for approval by the institution's ethics committee, and a positive opinion was obtained.

Surgical technique

**Figure 1:**
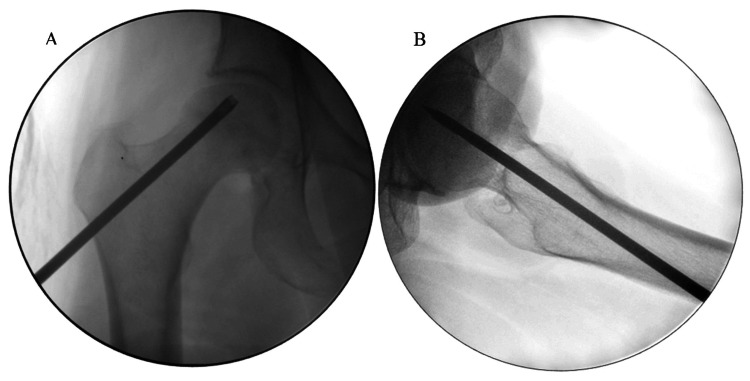
Intraoperative fluoroscopic images of femoral head core decompression A: anteroposterior view, B: lateral view

Patients are placed in dorsal decubitus on an orthopedic table, and the surgical fields are prepared after disinfecting the skin. The procedure begins with the collection of bone marrow aspirate by percutaneous puncture with a trocar at the anterior part of the iliac crest. The trocar is inserted to a depth of around 3 cm into the iliac medullary cavity, and two 30 mL syringes of aspirate are obtained. This aspirate is processed, filtered, and centrifuged until the bone marrow concentrate is obtained. Core decompression is then performed using a small incision on the lateral aspect of the thigh. Under fluoroscopic control, a 10 mm hollow cannula is used to pierce the lateral cortex of the femur and advance to the area of necrosis, allowing decompression. Around 20-30 mL of bone marrow concentrate is instilled directly into this area. The wound is closed, and a dressing is applied (Figure 1A, 1B).

## Results

Our series included 18 patients with a total of 24 affected hips. The vast majority (16 patients) were male, while only two were female. The average age of the patients at the time of decompression was 44 years (minimum: 35, maximum: 53).

Regarding risk factors, there was a clear association with tobacco smoking, which was present in 21 patients (16 hips). Additionally, alcohol consumption was present in six patients (eight hips), hypertension in four patients (four hips), dyslipidemia in four patients (four hips), and diabetes mellitus in two patients (two hips). No risk factors were identified in four patients (six hips) (Table 1).

**Table 1:** Demographic data, laterality of the lesion, and associated risk factors M: male, F: female

Age	Sex	Affected side	Risk factors
41	M	Bilateral	-
49	M	Right	Smoking + alcohol
53	M	Right	Smoking + hypertension
47	M	Left	Smoking + alcohol+ dyslipidemia
48	M	Bilateral	Smoking + alcohol
40	M	Left	Diabetes + dyslipidemia
53	M	Right	Smoking + hypertension
37	M	Bilateral	Smoking
37	F	Left	-
43	M	Bilateral	Smoking + alcohol
35	M	Right	Smoking
38	F	Right	-
56	M	Left	Smoking + diabetes
43	M	Bilateral	-
49	M	Left	Alcohol + hypertension + dyslipidemia
35	M	Right	Smoking + alcohol
44	M	Bilateral	Smoking
51	M	Left	Smoking + hypertension + dyslipidemia

According to the ARCO classification, we classified two hips as stage I, 18 as stage II, and four as stage III. The modified Kerboul angle measurements identified 16 hips in the low-risk prognostic group (angle: <190°), four hips in the moderate-risk group (angle: 190°-240°), and four hips in the high-risk group (angle: >240°). The Kim and Koo necrosis index identified 18 hips in grade A (necrosis index: ≤33%), six hips in grade B (necrosis index: 34%-66%), and none in group C (necrosis index: >66%) (Table 2).

**Table 2:** Staging (ARCO classification), osteonecrosis extension (Kerboul angle and Kim and Koo index) and final result obtained. ARCO: Association Research Circulation Osseous, R: right, L: left, S: success, F: failure

ARCO	Kerboul angle (°)	Kim and Koo (%)	Result
II R/II L	191°/235°	28.1%/42.4%	F
II	167°	21.1%	F
III	174°	22.3%	F
II	148°	16.8%	S
III R/II L	251°/180°	48.6%/24.9%	F/F
II	168°	21.2%	F
II	150°	17.2%	F
II	254°/138°	49.0%/14.3%	F/S
I	134°	14.1 %	S
III R/II L	205°/175°	32.4%/23.6%	F/F
II	144°	16.0%	S
I	129°	12.8%	S
II	169°	22.0%	F
II R/II L	243°/178°	45.5%/23.8%	F/F
II	172°	18.4%	F
II	142°	15.1%	S
II	235°/187°	39.1%/26.8%	F/F
III	256°	49.4%	F

**Figure 2:**
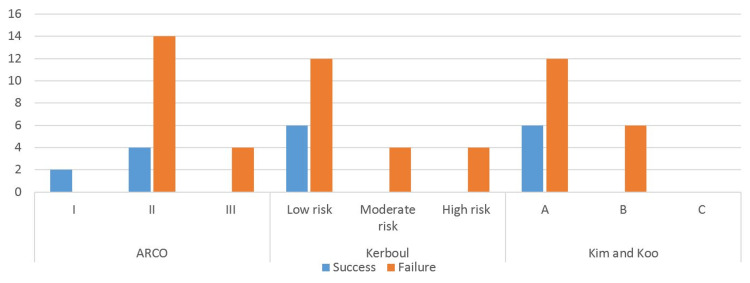
Distribution of successful and failed interventions according to staging (ARCO classification) and degree of lesion extension (Kerboul angle and Kim and Koo index) ARCO: Association Research Circulation Osseous

The Postel-Merle d'Aubigné functional score was used in patients with a favorable evolution (no need for arthroplasty), and a mean value of 16.7 was obtained, with most patients reporting only a mild degree of pain/discomfort. The average survival time after core decompression in the overall series was 14 months. The outcome was favorable in six hips or 25% of cases. Conversely, salvage arthroplasty was necessary in 18 hips, corresponding to 75% of cases (Figure 2).

## Discussion

Regarding the epidemiological data in the series, in addition to the lower incidence presented, the female sex proved to be a good prognostic factor. Smoking was the main factor identified as contributing to AVN incidence, but there were no significant differences in prognosis between the failure group and the success group.

There is still no consensus on an ideal treatment protocol for patients with AVN in the pre-collapse stage of the femoral head, but early intervention is fundamental for successful results in joint preservation procedures. According to the literature, core decompression is the most commonly used treatment in the early stages of the disease and is only recommended in these cases [1,3,7]. Our series only included two stage I patients, a low number that may reflect the initial difficulty in diagnosis. At this stage, patients are often asymptomatic or have mild complaints, which can lead to a delay in diagnosis and treatment. In these two patients, treatment was successful, and the clinical results were favorable. For stage II patients, the treatment failure rate was 78%. In stage III patients, treatment failed in 100% of cases. These poor results coincide with previous studies that have consistently observed poor clinical outcomes in this group, with several authors therefore considering stage III to be a contraindication to core decompression [8].

A review of the results of other published series reveals great variability in the clinical failure rates found, with values ranging from 4% to 75% in stage I and from 10% to 86% in stage II [8]. This variability suggests that parameters other than the ARCO stage should be taken into consideration to better understand prognosis and treatment efficacy. In our series, larger volume lesions on MRI (with a modified Kerboul angle greater than 150° and a Koo index greater than 20%) had a significantly higher risk of treatment failure (p < 0.05). Several authors consider the size of the lesion to be an essential prognostic factor [3,9]. Previous studies have suggested that a Koo index > 40% should be an absolute contraindication to core decompression. Furthermore, a Koo index between 30% and 40% is considered a poor prognostic factor [8].

The use of biological agents to enhance core decompression and facilitate the remodeling of necrotic areas, with osteogenic and/or osteoinductive factors, could in theory have the potential to produce better results in larger lesions. In fact, some studies have shown differences in the time until the collapse of the femoral head and a reduction in the size of the necrotic lesion [3]. However, in our study, the combined use of BMAC did not seem to offer any additional benefits in larger lesions. In the core decompression success group, the average Postel-Merle d'Aubigné functional score of 16.7 indicates that these patients had good results regarding pain, joint mobility, and walking functionality. The majority of patients reported some degree of discomfort, but without preventing them from carrying out daily life activities. These results coincide with previous publications, proving core decompression with bone marrow injection to have excellent functional outcomes [10].

Our study has some limitations that should be taken into consideration: it is a retrospective study, meaning that the data was collected "a posteriori", possibly leading to incomplete or incorrect data; the sample size was small, with only 18 patients, limiting the generalization of the study's results; the location of the area of necrosis on the femoral head was not taken into account in the assessment, which can be an important factor affecting the patient's prognosis; and errors in the measurement of angles and volumes by the two observers are to be expected, as high-precision digital quantification methods were not used, making the results less reliable.

## Conclusions

According to the literature, early-stage AVN of the femoral head is considered the ideal indication for core decompression, but this isolated aspect is no guarantee of surgical success. In this study, patients with smaller preoperative lesions (modified Kerboul angle < 150° and Koo index < 20%) had a more favorable outcome compared to patients with larger lesions. As such, our results seem to confirm the importance of lesion size as a prognostic factor in the treatment of AVN.
